# Designing an educational intervention to prevent excessive gestational weight gain: a protocol for a randomized controlled trial

**DOI:** 10.1186/s12978-019-0696-7

**Published:** 2019-03-12

**Authors:** Hossein Shahnazi, Nafiseh Abdolalian, Ashraf Kazemi, Akbar Hassanzadeh

**Affiliations:** 1 Department of Health Education and Promotion, School of Health, Isfahan University of Medical Sciences, Isfahan, Iran; 2 Nursing and Midwifery Care Research Center, School of Nursing and Midwifery, Isfahan University of Medical Science, Isfahan, Iran; 3 Department of Epidemiology and Biostatistics, School of Health, Isfahan University of Medical Sciences, Isfahan, Iran

**Keywords:** Education, Obesity, Pregnancy, Weigh gain

## Abstract

**Background:**

Pregnancy as one of the critical stages of life carries a high risk to the health of pregnant women. The amount of weight gained during pregnancy can affect the woman and her infant health immediately or in the future. The present study is conducted to design and explore the effectiveness of an educational intervention based on health belief model (HBM) to preventing excessive gestational weight gain (GWG).

**Methods:**

This research-based planning is designed in three phases and will be conducted on pregnant women in first trimester. In the first phase of this randomized controlled trial study, body mass index (BMI), the level of knowledge and the level of the HBM constructs will be measured using a questionnaire. The HBM questionnaire is designed based on a literature review and experts opinions. In the next phase the educational program content will be designed based on the results of the first phase of the study on the level of women’s knowledge, and HBM constructs as well as a literature review and experts opinions. The intervention will be designed in four training sessions about the importance of behaviors, especially physical activity and nutrition, in the prevention of excessive weight gain during pregnancy. The tired phase includes the implementation of educational intervention with two intervention and control groups. The efficacy of the program will be evaluated by measuring the level of the knowledge, HBM constructs and women’s weight gain during pregnancy in the second and third trimesters. Appreciate weight gain will be considered according to the BMI in first trimester.

**Discussion:**

The present study will provide strong information regarding the effetiness of the HBM and appropriate framework to develop educational interventions together with enhancing pregnant women’s knowledge and belief toward weight management behaviors.

**Trial registration:**

Registration of this randomized control trial has been completed with the Iranian Registry of Clinical Trials, IRCT20180703040325N1. Date of registration: 2018-08-20.

## Plain English summary

Pregnancy as one of the critical stages of life carries a high risk to the health of pregnant women. The amount of weight gained during pregnancy can affect the woman and her infant health immediately or in the future. The present study is conducted to design and explore the effectiveness of an educational intervention based on health belief model (HBM) to preventing excessive gestational weight gain (GWG). This research-based planning is designed in three phases and will be conducted on pregnant women in first trimester. In the first phase of this randomized controlled trial study, body mass index (BMI), the level of knowledge and the level of the HBM constructs will be measured using a questionnaire. The HBM questionnaire is designed based on a literature review and experts opinions. In the next phase the educational program content will be designed based on the results of the first phase of the study on the level of women’s knowledge, and HBM constructs as well as a literature review and experts opinions. The intervention will be designed in four training sessions about the importance of behaviors, especially physical activity and nutrition, in the prevention of excessive weight gain during pregnancy. The tired phase includes the implementation of educational intervention with two intervention and control groups. The efficacy of the program will be evaluated by measuring the level of the knowledge, HBM constructs and women’s weight gain during pregnancy in the second and third trimesters. Appreciate weight gain will be considered according to the BMI in first trimester. The present study will provide strong information regarding the effetiness of the HBM and appropriate framework to develop educational interventions together with enhancing pregnant women’s knowledge and belief toward weight management behaviors.

## Background

Today, excessive gestational weight gain (GWG) is considered one of the common concerns of pregnant women. It carries a high risk of pregnancy, puerperal, and neonatal complications [1–3]. To minimize the negative health consequences of excessive weight gain for both the pregnant women and the fetus, the Institute of Medicine guidelines have long provided ranges of recommended weight gain for pregnant women on the basis of their pre-pregnancy body mass index (BMI) [4, 5]. Excessive BMI is an important reason for lifelong obesity among women [6] and their children [7]. In addition, many studies indicated that pregnant women have a low intake of fruit, vegetables, and dairy products and a high intake of sweets and pastries [8]. It was also reported that the decreased consumption of fruit and vegetables, high energy intake [9], and sedentary lifestyle (i.e. less than two and a half hours of physical activity during a week) are associated with excessive GWG [10].

Studies have also proven that interventions aiming for a lifestyle change lead to a reduction in excessive GWG [11]. Some reasons for excessive GWG include lack of knowledge about excessive GWG and its risks, lack of access to adequate and transparent nutritional information [9, 12], wrong information about pregnancy diet [13], perceived risk of pregnancy challenges and physical activity to fetal health [12, 14], lack of perceived self-efficacy to gestational weight management [1, 15], and failure of healthcare providers to provide adequate information about appropriate prenatal physical exercise and dietary intake [14, 16].

The constructs of the HBM are always considered in pregnancy because both behavior change model for preventing weight gain [17] and an effective model for identifying behavior change factors [18]. According to the HBM, individuals perceive themselves to be susceptible to a negative health outcome when they perceive it to be severe. Then, they perceive the benefits of adopting a healthy behavior to be more than its barriers. Hence, individuals decide to adopt that healthy behavior. In fact, the core constructs of adopting a healthy behavior are perceived susceptibility, perceived severity, perceived benefits, perceived barriers, perceived self-efficacy, and cues to action [19]. Educational interventions highlight the positive effect of education on improving weight-gain behaviors, the level of physical activity, and the type of pregnancy diet [20, 21]. Model-based education can decrease the logistical constraints and burden by weight management interventions [22]. Therefore, the purpose of this study was to design an educational program about optimal GWG among pregnant women.

## Methods/ design

This research-based planning is designed in three phases and will be conducted on 100 first trimester pregnant women who will refer to health centers in Isfahan- Iran.

The sample size was calculated the following equation. The sample size for each group will be 50 (α = 0.05, a power of 90%, and a standardized effect size of 0.65).

$$ n=\frac{2{\left({z}_{1-\alpha /2}+{z}_{1-\beta}\right)}^2}{\Delta^2}+\frac{Z_{1-\alpha /2}^2}{2}=\frac{2{\left(1.96+1.28\right)}^2}{0.65^2}+\frac{1.96^2}{2}\cong 50 $$

The inclusion criteria will be a willingness to participate in the study, the age range of 18–35 years, pre-pregnancy BMI between 18.5 to 25 kg/m^2^, having no diagnosed psychological disorder such as anxiety, depression, and obsessive-compulsive disorder, gestational age 11–12 weeks, and singleton pregnancy. The exclusion criteria include suffering from pregnancy complications including polyhydramnios, eclampsia and preeclampsia.

In the first phase of this randomized controlled trial study, body mass index (BMI), the level of knowledge and the level of the HBM constructs will be measured using a questionnaire. The HBM questionnaire is designed based on a literature review and experts opinions.

### Sampling

In this study, subjects will be selected by proportional stratified random sampling. Thus, in order to consider the socioeconomic status of pregnant women in the study, first, all districts in Isfahan will be categorized into five sections according to municipality divisions, namely northern, southern, western, eastern, and central. Two health centers will be randomly selected from each district and sampling will be carried out. Totally, 100 pregnant women from 10 health centers will be enrolled in the study.

### Measures

After obtaining written informed consent and receiving the researcher’s necessary guidance the BMI of the subjects will be measured. Also, the levels of the knowledge and HBM constructs will be assessed using relevant questionnaires.

### Data-collection tools

To collect data, a researcher-designed questionnaire based on the HBM constructs will be used. The questionnaire has three sections as follows. The first section consisted of demographic questions. The second section is about knowledge regarding excessive GWG, which comprised 11 items. The response options are “Yes,” “No,” and “Do not know,” which were scored 2, 0, and 1, respectively with a range from 0 to 22. The third section relates to the perceived susceptibility, for example “excessive GWG is not harmful among women who are not sick”, perceived barriers, for example, “it is difficult to control pregnancy weight gain within a normal range”, perceived self-efficacy, for example, “I am certain I can prevent excessive GWG, if I go on a well-balanced diet”, and cues to action, for example, “the role of important persons such as spouse in managing GWG”.

To evaluate perceived susceptibility, seven items are designed with a range between 7 and 35. Furthermore, five items were developed to assess perceived barriers and perceived self-efficacy with a range between 5 and 25. A five-point Likert scale (“agree,” “somewhat agree,” “do not know”, “somewhat disagree,” and “disagree”) are used for all the constructs. In addition, in order to assess cues to action, seven items are designed and they are scored using a five-point Likert scale from very high to very low with a range between 7 and 35.

In all items of the questionnaire (knowledge and HBM constructs), reverse scoring is done for negative questions. Higher scores for all the items, except perceived barriers, indicate a better condition.

The validity of the questionnaire was evaluated by a panel of experts. The designed questionnaire was sent to six professors of health education and midwifery. After submitting their comments, the validity of the questionnaire was approved. The content validity ratio (0.87) and the content validity index (0.81) were used to assess the validity of the questionnaire. The test-retest method, which shows the repeatability of an index, was used to evaluate the reliability of the questionnaire. The validated questionnaire was handed out to 20 pregnant women. After two weeks, they were asked to re-complete the questionnaire. Given the findings, none of the items was removed. The Cronbach’s alpha coefficient was computed for each construct (knowledge = 0.708, perceived susceptibility = 0.787, perceived barriers = 0.723, perceived self-efficacy = 0.733, and cues to action = 0.711).

### Intervention program

In the next phase the educational program content will be designed based on the results of the first phase of the study on the level of women’s knowledge, and HBM constructs as well as a literature review and experts opinions. The intervention will be designed in four one-hour training sessions about the importance of behaviors, especially physical activity and nutrition, in the prevention of excessive weight gain during pregnancy.

The educational content will be about the significance of a well-balanced diet and physical activity to prevent pregnancy overweight. These sessions will be held by a health education specialist together with the collaboration of a nutritionist and a physical medicine doctor. The participants will be provided with question-and-answer sessions, group discussions, role-play, verbal encouragement, and educational videos. These techniques and tools aimed to modify the HBM constructs toward optimal GWG. In the last session, women’s husbands, as cues to action, accompanied them. Their husbands will be asked to help them with their self-care behaviors to manage weight (Table 1).

**Table 1:** Educational goals, strategies and examples of training sessions for pregnant women

Educational goal	Strategies	Examples
Knowledge	Lectures, written materials provided in print, individual counseling	Increasing knowledge about the usefulness of healthy diet and physical activity, duration of physical activity, familiar to the change of nutritional needs in pregnancy, individual Permissible weight gain according to the Institute of Medicine’s protocol
Perceived susceptibility	Presentation of epidemiological studies	Using graphs to describing national statistics of the fetal, maternal and pregnancy complication with regard to excessive GWG
Self-efficacy	-Break the goal into smaller stages - Practical role model -Verbal encourage	Diet compliance during one day Substitute fruit instead of cake and cookies as snack Do exercise in 10 min at a time Invitation of a mother with a good fitness to talk about successful experiences related to physical activity in previous pregnancy Weighting the participants end of the class and chat about who achieved to appropriate weight gain
Perceived Barrier	Discussion to remove and Correct of the false beliefs about physical activity and nutrition in pregnancy	Not eating as much as 2 people Exercise won’t harm the baby Doing Stretching exercise practically in a short time at the class
Cue to action	Helping from other family member	Sending message about daily walking Spouse involvement to recall the correct nutrition

### Implementation

The tired phase includes the implementation of educational intervention. In this phase the participants will be divided into the intervention and the control group using randomized blocks with block sizes of 4 and 6 and allocation ratio of 1:1 and stratification by health centers.

In order to motivate the registered pregnant women to participate in the study, they will call and informed about the objectives of the study. The designed intervention program will be implemented for intervention group and the control group will be attended regularly scheduled counseling with their prenatal care providers, which typically occurred monthly.

### Evaluation

The efficacy of the program will be evaluated by measuring the level of the knowledge, HBM constructs and women’s weight gain during pregnancy in the second and third trimesters. Appreciate weight gain will be considered according to the BMI in first trimester.

### Data analysis

The SPSS V.19 software will be used to analyze the data. The normality of the variables will be examined using the Kolmogorov-Smirnov test. The independent t-test and the repeated measures ANOVA will be used to compare the HBM constructs with the pre- and post-intervention weight. Chi-square test will be also used to compare GWG in different time-points and demographic variables between the two groups.

## Discussion

Generally, the health belief theories demonstrated that individuals with low perceived susceptibility deny the possibility of suffering from a health condition or disease, those with average perceived susceptibility consider themselves susceptible to a health condition, and those with high perceived susceptibility perceive the real risk of suffering from a health condition [23]. Some studies showed the effect of educational interventions on increasing perceived susceptibility to dietary behaviors among pregnant women [24, 25]. Considering the hypothesis of the present study individuals’ perceived susceptibility could be increased by designing and implementing educational interventions. The increased perceived susceptibility increases risk perception and, accordingly, the likelihood of performing healthy behaviors.

Drawing on group discussions between participants and recommending procedures to have a healthy diet, the present study attempts to reduce perceived barriers to healthy diet and physical activity among pregnant women. Common cultural beliefs in Iran about the risk of physical activity on fetal health have led to observe a low level of physical activity among Iranian pregnant women [26]. Numerous piece of research demonstrated that the most important intrapersonal barriers to women overcoming excessive GWG include anxiety over fetal health, feeling tired after doing physical activity, others’ advice against gestational physical activity, time constraints for physical exercise and healthy eating, easy access to unhealthy food, lack of knowledge and skill, and cravings [27].

Since particular strategies will be employed for enhancing self-efficacy in the present study [28], the educational intervention may have a positive effect on the self-efficacy of the intervention group. This research based program may empower the pregnant women to perform proper dietary behaviors using behavior patterns, giving verbal encouragement, and breaking down a complex behavior into simple practical and accessible behaviors.

Self-efficacy is one’s belief in one’s ability to perform recommended and adaptive behaviors well by overcoming complex behavior. Low self-efficacy is one of the effective psychological factors in excessive GWG [29]. It is belief that the appreciate interventions training which increases self-efficacy also enhances the individual’s ability to change behaviors for managing weight and performing physical activity [30]. Considering some studies, increased self-efficacy significantly affected weight loss [31].

We suppose this research based program has capacity to integrate into the professionally health care guidelines and HBM-based educational intervention could affect cognitive factors related to excessive weight gain during pregnancy.

## Abbreviations

BMI: Body mass index
HBM: Health Belief Model, GWG: gestational weight gain
